# Involuntary psychiatric treatment in Reggio Emilia: local findings from the SIEP multicenter study

**DOI:** 10.3389/fpsyt.2026.1739274

**Published:** 2026-02-02

**Authors:** Luca Pingani, Giulia Dragani, Nadia Magnani, Mattia Marchi, Anna Maria Nasi, Fabrizio Starace, Gian Maria Galeazzi

**Affiliations:** 1 Department of Biomedical, Metabolic and Neural Sciences, University of Modena and Reggio Emilia, Modena, Italy; 2 Department of Mental Health and Pathological Addictions, Azienda USL–IRCCS di Reggio Emilia, Reggio Emilia, Italy; 3 Department of Adult Mental Health, Grosseto Unit, Azienda USL Toscana Sud Est, Grosseto, Italy; 4 Department of Mental Health and Pathological Addictions (DSMDP), Azienda Sanitaria Locale TO5, Turin, Italy

**Keywords:** coercion, commitment of mentally ill, health services research, involuntary psychiatric treatment, physical restraint, recovery-oriented care, schizophrenia spectrum and other psychotic disorders

## Abstract

**Introduction:**

Involuntary psychiatric treatment (IPT) remains a controversial practice, raising clinical, ethical, and organizational concerns. In Italy, despite legal safeguards, substantial variability persists across regions, reflecting differences in clinical presentation, service organization, and staff attitudes. As part of the national multicenter project promoted by the Italian Society of Psychiatric Epidemiology (SIEP), this study reports findings from the Reggio Emilia site, exploring factors associated with IPT duration, renewal, and the use of mechanical restraint.

**Methods:**

All adult patients admitted under IPT to the Psychiatric Diagnosis and Treatment Unit of Reggio Emilia between March 2023 and March 2024 were included (N = 214). Data were collected with standardized SIEP forms and analyzed using descriptive and inferential statistics.

**Results:**

The mean duration of IPT was 7.4 days (SD = 3.8). A history of prior IPT was strongly associated with renewal (37.7% vs. 11.9%, p < 0.001). First-ever contact and conversions from voluntary admission were not significant. Schizophrenia and delusional disorders were associated with longer hospitalizations (p < 0.01). Male patients were more often mechanically restrained (10.8% vs. 1.1%, p = 0.006), though duration did not differ by gender. Differences in IPT duration across validation settings were observed; however, *post hoc* analyses indicated that these were primarily driven by a small subgroup of cases validated in residual or atypical services, while emergency, inpatient, and community pathways showed broadly comparable durations.

**Discussion:**

Overall, prior coercion history and organizational factors appeared to play a more prominent role than demographic characteristics in shaping IPT-related outcomes. These findings highlight the importance of strengthening community-based alternatives, early intervention strategies, and bias-aware staff training to reduce reliance on coercive practices.

## 1 Introduction

Law No. 180 of May 13, 1978—commonly referred to as the “Basaglia Law”—revolutionized psychiatric care in Italy and became a global reference for deinstitutionalization and rights-based mental health reforms. Named after psychiatrist Franco Basaglia, leader of the Italian mental health reform movement, the law mandated the closure of psychiatric hospitals, the suspension of new admissions to asylums, and the progressive construction of a community-based mental health system rooted in voluntary care, rehabilitation, and the protection of civil rights (1). It was the first national law to abolish the asylum system, asserting that treatment should occur in general hospitals and community services, and that coercive practices be minimized and subjected to strict judicial oversight.

Law 180 was not only a legal reform but a cultural and ethical statement: it redefined mental illness not as a reason for segregation, but as a condition to be addressed in the open community through multidisciplinary and integrated care. It was later incorporated into Law 833/1978, which established Italy’s National Health Service (2). The impact of Law 180 extended beyond Italian borders, inspiring rights–based approaches and deinstitutionalization movements worldwide. Nevertheless, the law continues to generate debate regarding its long-term implementation, especially concerning the availability of community resources and crisis management strategies (3).

Involuntary psychiatric treatment (IPT), or “Trattamento Sanitario Obbligatorio (TSO)” in Italian law, refers to psychiatric hospitalization carried out without the patient’s consent, under the specific legal conditions set forth by Law 833/1978. According to Article 33 of this law, IPT can be enacted only when three criteria are simultaneously met: (1) the presence of psychic alterations of a degree and nature which require urgent intervention; (2) the patient’s refusal of treatment; and (3) the unavailability of appropriate treatment alternatives to inpatient treatment. IPT must be proposed by at least two medical doctors, at least one of which belonging to National Health, authorized by the mayor of the locality where the IPT take place, and confirmed by a judge within 48 hours. Its initial duration is limited to seven days, with the possibility of weekly renewals under judicial monitoring (2). In the Italian legal framework, IPT primarily refers to involuntary psychiatric hospitalization and treatment which includes most commonly also pharmacological compulsory treatment. However, during an IPT episode, additional restrictive measures may occur in clinical practice, including mechanical restraint and seclusion (this latter extremely rare in Italy). These measures are not legally synonymous with IPT, but they are often operationally intertwined and are therefore discussed within the broader spectrum of coercive practices.

In practice, IPT may be considered when an acute psychiatric crisis causes mental symptoms of such intensity which require urgent therapeutic interventions, the person refuses such interventions, and no feasible community-based alternatives can ensure safety and continuity of treatment. In such scenarios, IPT is intended as a time-limited measure under judicial oversight, aimed at restoring clinical stability and enabling a return to voluntary, least-restrictive care.

While IPT may be necessary in some acute situations, it remains a controversial intervention due to its ethical implications and potential for misuse. Numerous studies have shown that coercion can negatively impact patients’ trust in services, therapeutic relationships, and long-term outcomes (4, 5).

Coercion can be conceptualized along at least two dimensions. Perceived coercion refers to the subjective experience of feeling pressured, threatened, or deprived of choice during care, regardless of formal legal status. By contrast, wrongful or unethical coercion refers to coercive actions that exceed clinical necessity or proportionality, violate procedural safeguards, or constitute misuse of power, potentially amounting to rights violations. IPT may be experienced as coercive even when legally justified, and this perceived coercion can affect trust, engagement, and future help-seeking.

Moreover, IPT is associated with other restrictive measures such as mechanical restraint, forced administration of medication, and seclusion, forming part of a broader spectrum of coercive practices (6, 7). For these reasons, IPT is often considered a marker of systemic failure—used when preventive, voluntary, and community-based interventions are unavailable, insufficient, or delayed.

Despite its legal safeguards and emphasis on least restrictive care, Italy still registers a significant number of involuntary psychiatric hospitalizations. According to the latest Mental Health Report from the Italian Ministry of Health, in 2023 there were 4,879 IPT cases nationwide, representing 5.7% of all psychiatric admissions. The average national rate stands at 1.0 IPT per 10,000 inhabitants, but with remarkable interregional variation—from 0.0 in Basilicata to 2.6 in Umbria. Such variability suggests that organizational, cultural, and policy differences among regions may heavily influence the implementation of IPT (8). However, the reliability of these data has been repeatedly questioned. Not all regions regularly transmit complete data to the national mental health information system, and certain episodes—such as involuntary admissions occurring while a patient is already hospitalized on a voluntary basis—are often underreported. This has led experts to highlight the risk of systematic underestimation, as recently emphasized in national debates and advocacy efforts, including those promoted by Starace and colleagues on the need for more transparent and comprehensive reporting (9).

International comparisons show even more pronounced differences. A major comparative study by Rains et al. (10) across 22 countries found IPT rates ranging from less than 15 per 100,000 inhabitants in Italy to over 280 in Austria. The same study concluded that legal frameworks alone do not account for such disparities. Rather, factors such as the availability of psychiatric beds, national income level, mental health workforce capacity, and cultural attitudes toward coercion all play a role. In high-income countries, the average IPT rate was 106.4 per 100,000 people, with significant outliers on both extremes.

A recent paper highlighted that the implementation of coercive practices varies widely even within countries and is strongly shaped by historical, cultural, and organizational contexts rather than clinical need (11). Moreover, according to the World Health Organization (12), coercion—including IPT—is overused globally, especially in contexts where community-based services and patient-centered crisis interventions are underdeveloped. Consequently, international guidelines now emphasize the development of alternatives to IPT and other coercive practices, urging countries to adopt recovery-oriented and rights-based models of care.

A growing body of literature has identified a complex array of clinical, demographic, organizational, and cultural factors that increase the likelihood of IPT. Among the most consistent clinical predictors are diagnoses within the schizophrenia spectrum and delusional disorders, which are associated with both a higher incidence and longer duration of compulsory hospitalization (13). Sociodemographic variables—such as being male, single, unemployed, or belonging to minority groups—have consistently been associated with increased IPT risk (13). Research has emphasized that not only immigrant status but also belonging to racial and ethnic minorities (e.g., BAME populations) or other marginalized groups exposed to discrimination may significantly contribute to higher rates of IPT (14).

In addition, previous episodes of coercion strongly predict future IPT (15), while substance use disorders and lack of family or social support have also been identified as relevant risk factors in systematic reviews (16).

From an organizational perspective, IPT is more prevalent in systems lacking early intervention services, continuity of care, and community-based crisis alternatives—while initiatives such as open-door policies, peer-support, and patient-controlled admissions have been proposed as effective countermeasures (17–19). Equally important are staff attitudes, which significantly shape the application of coercion: professionals who perceive coercive interventions as necessary or protective are more likely to apply them, whereas those endorsing recovery-oriented approaches tend to rely on de-escalation and consensual strategies (20, 21).

The consequences of IPT are profound and multifaceted. On an individual level, involuntary hospitalization is frequently experienced as traumatic, leading to feelings of fear, humiliation, and powerlessness (13, 22). It can undermine the therapeutic relationship, diminish trust in mental health services, and discourage future help-seeking behavior (23). At the systemic level, the frequent use of coercion may reflect service failure rather than clinical necessity, highlighting critical gaps in prevention, accessibility, and person-centered care (12). These findings underscore the importance of addressing not only clinical need, but also the broader structural and cultural drivers of IPT, in efforts to promote more ethical, effective, and rights-based mental health care.

The Italian Society of Psychiatric Epidemiology (SIEP) launched a national multicenter study to examine clinical, demographic, organizational, and attitudinal factors influencing IPT in Italian mental health services. The project also investigates how professional beliefs, and institutional settings affect both the use and duration of compulsory interventions.

This paper reports findings from the Reggio Emilia site, where data was collected over 12 months from all adult patients admitted under IPT to the Psychiatric Diagnosis and Treatment Unit (SPDC) of Correggio, serving a catchment area of about 530,000 residents.

Based on prior literature, we formulated hypotheses across three domains:

- Service engagement and clinical history: first-ever patients would have longer IPTs than those already in care; previous IPT would predict renewal; conversions from voluntary to compulsory admissions would result in shorter stays.
- Diagnostic and clinical characteristics: schizophrenia and delusional disorders would be associated with longer IPTs than other diagnoses.
- Gender differences and care pathways: male patients would be more frequently restrained, female patients would have shorter IPTs, and IPTs validated in emergency departments would be shorter than those validated in wards or community services.

## 2 Methods

### 2.1 Study design and context

This study is part of a national multicenter observational project promoted by the Italian Society of Psychiatric Epidemiology (SIEP), aimed at identifying clinical, demographic, and organizational factors associated with IPT across Italian mental health services. The current analysis focuses on data collected at the Reggio Emilia site, located in Northern Italy, under the coordination of the Department of Mental Health and Substance Use of the Azienda USL–IRCCS of Reggio Emilia. Within the department, there is a single Psychiatric Diagnosis and Treatment Unit, located in the town of Correggio and comprising 16 beds. This unit is specifically designated to admit and treat all patients undergoing IPT across the province, which has a population of approximately 530,000 inhabitants. Data collection at this site took place over a 12-month period, from March 20th, 2023, to March 19th, 2024.

### 2.2 Participants and inclusion criteria

All patients admitted to the Psychiatric Diagnosis and Treatment Unit under IPT during the study period were included in the analysis. Only adult patients aged 18 years or older were eligible.

### 2.3 Data collection procedures

Data were gathered using standardized event forms developed by the SIEP coordinating group. These forms were completed by trained clinical personnel immediately following each IPT admission. Collected information included sociodemographic data (age, gender), clinical data (diagnosis categorized as schizophrenia spectrum disorders, bipolar disorder, personality disorders, or other), toxicological screening results at admission, and whether mechanical restraint was applied during hospitalization.

In addition, data were collected on service-related factors, including whether the patient was already in contact with a Community Mental Health Center (CMHC), whether the hospitalization represented the first-ever contact with mental health services, and whether the IPT followed a voluntary admission. Legal and procedural variables were also documented, such as the source and setting of IPT validation (e.g., emergency department, inpatient unit, or territorial services), whether the IPT was renewed after the initial seven days, and whether the patient filed a legal appeal. Among the variables collected, the duration of the IPT in days was also recorded. All data were anonymized and transferred to a centralized national database for analysis.

### 2.4 Statistical analysis

All statistical analyses were conducted using IBM SPSS Statistics 27 (24) and JASP version 0.19.3 (25). Descriptive statistics were computed for all variables, including frequencies and percentages for categorical variables, and means and standard deviations or medians and interquartile ranges for continuous variables, depending on their distribution.

The normality of distribution for continuous variables, particularly IPT duration, was assessed using the Kolmogorov–Smirnov test, along with visual inspection of histograms and Q–Q plots. If the assumption of normality was met, comparisons between two independent groups were conducted using independent samples t-tests, while comparisons across more than two groups were analyzed using one-way ANOVA. In the case of non-normally distributed variables, Mann–Whitney U tests and Kruskal–Wallis tests were applied. Where appropriate, Dunn’s *post-hoc* tests with Bonferroni correction were used to explore pairwise differences.

Associations between categorical variables were analyzed using the Chi-square test of independence, or Fisher’s exact test when expected frequencies were below the recommended threshold. All tests were two-tailed, and statistical significance was set at p<0.05.

Given the observational nature of the study and the limited set of covariates available in the SIEP event forms, analyses were conducted as exploratory, hypothesis-guided comparisons rather than as predictive modelling. No single primary outcome was predefined; instead, we examined three pre-specified IPT-related outcomes: duration (days), renewal (yes/no), and mechanical restraint (yes/no).

## 3 Results

### 3.1 Clinical and diagnostic profile

Between March 20, 2023, and March 19, 2024, all patients who underwent involuntary psychiatric hospitalization (IPT) during the study period were enrolled, resulting in a total sample of 214 individuals admitted under IPT to the Psychiatric Diagnosis and Treatment Unit. The sample comprised 112 males (52.3%), with the most represented age group being between 41 and 60 years (39.7%). Regarding psychiatric diagnoses, the most frequent were schizophrenia spectrum disorders (53.7%), followed by bipolar disorder (14%). Other diagnoses (18.7%), including major depressive disorder and substance-induced psychotic disorders. In addition, toxicological screening at admission indicated recent substance use in 20.1% of patients. For 67.3% of the patients involved (N = 144), this was their first IPT since the beginning of the study.

### 3.2 Service engagement and legal pathway

At the time of admission, 65.4% of patients were already under the care of a Community Mental Health Center (CMHC), while 35 were first-time contacts with mental health services. Additionally, 8.4% of the sample had initially been voluntarily admitted, with their status subsequently converted to involuntary hospitalization.

For 37 patients, the IPT was renewed at least once beyond the initial seven-day period. Only one patient filed an appeal against the TSO during the study period. The majority of IPTs were validated by community psychiatric services (N = 95) and emergency departments (N = 70).

All these results are presented in **Table 1**.

**Table 1:** Sociodemographic, clinical, and care-pathway characteristics of patients admitted under involuntary psychiatric treatment (IPT).

Variables		Absolute frequency	Percentage frequency
Sex	Male	112	52.3
	Female	89	41.6
	Missing data	13	6.1
Age	18 to 24 years	26	12.1
	25 to 40 years	23	10.7
	41 to 60 years	85	39.7
	Over 60 years	67	31.3
	Missing data	13	6.1
Primary diagnosis	Schizophrenia spectrum disorders	115	53.7
	Bipolar disorder	30	14
	Personality disorders	17	7.9
	Other diagnoses	40	18.7
	Missing data	12	5.6
If the patient underwent blood alcohol or toxicology screening at admission, was the result positive?	No	88	41.1
	Yes	43	20.1
	Not performed	67	31.3
	Missing data	16	7.5
Has the patient ever been admitted under IPT before?	No	144	67.3
	Yes	53	24.8
	Missing data	17	7.9
Was the involuntary hospitalization (TSO) renewed at least once after the initial seven-day period?	No	163	76.2
	Yes	37	17.3
	Missing data	14	6.5
Was the patient already under the care of the Community Mental Health Center associated with the Psychiatric Diagnosis and Treatment Unit?	No	61	28.5
	Yes	140	65.4
	Missing data	13	6.1
Was the patient not yet under the care of the CMHC(s) associated with the SPDC, but part of the SPDC’s catchment area (“first ever” patients, i.e., first-time contacts with mental health services)?”	No	23	37.1
	Yes	35	58.1
	Missing data	3	4.8
Did the patient appeal the IPT?	No	198	92.5
	Yes	1	0.5
	Missing data	15	7
Was the IPT converted from a voluntary admission?	No	180	84.1
	Yes	18	8.4
	Missing data	16	7.5
Where was the IPT validated?	Community psychiatric services	95	44.4
	Emergency Department	70	32.7
	Psychiatric Diagnosis and Treatment Unit	22	10.3
	Psychiatric Consultation Service	4	1.9
	Other services	8	3.7
	Missing data	15	7

Percentages may not total 100 due to rounding.

### 3.3 Coercive measures and clinical outcome

The average duration of IPT was 7.4 days (SD= ± 3.8), with a minimum of 1 day and a maximum of 28 days. Mechanical restraint was used in 13 cases, corresponding to 6.1% of the sample, while it was not applied in 87.4% of cases. These findings are summarized in **Table 2**.

**Table 2:** Duration of involuntary psychiatric treatment (IPT) and use of mechanical restraint during hospitalization.

Variables		Mean	SD	Minimum	Maximum
Duration of IPT (days)		7.4	±3.8	1	28
Variables		Absolute frequency		Percentage frequency	
Episodes of mechanical restraint during IPT	No	187		87.4	
	Yes	13		6.1	
	Missing data	14		6.5	

### 3.4 Results of hypothesis testing

#### 3.4.1 Service engagement and clinical history

A Mann–Whitney U test showed no significant differences in the duration of IPT between patients at their first contacts with mental health services (“first ever”) and those already engaged with a CMHC (U = 321.000, p=0.17). The mean duration was 5.0 days (SD ± 2.4) for first-ever patients and 6.7 days (SD=± 3.3) for those previously in care, but the difference did not reach statistical significance.

In contrast, a history of prior IPT was strongly associated with renewal. Among patients with at least one previous IPT, 37.7% experienced renewal of the current episode, compared to 11.9% of those undergoing their first IPT (χ²(1)=16.87, p<0.001).

Finally, no significant differences were found in the duration of hospitalization between admission that started as voluntary and were subsequently converted to IPT (mean 7.2 days, SD = ± 2.9) and those admitted directly under IPT (mean 7.4 days, SD= ± 3.9; U = 1634.000, p=0.88).

#### 3.4.2 Diagnostic and clinical characteristics

A Kruskal–Wallis test showed that the duration of IPT significantly differed across diagnostic groups (χ²(3)=15.49, p=0.001). Patients diagnosed with schizophrenia or delusional disorders had the longest average duration (mean 8 days, SD= ± 3.9), followed by those with bipolar disorder (mean 6.9 days, SD= ± 3.4), other diagnoses (mean 6.4 days, SD= ± 3.8), and severe personality disorders (mean 5.9 days, SD= ± 3.1). *Post hoc* analyses indicated that patients with schizophrenia or delusional disorders were hospitalized significantly longer than those with severe personality disorders (z=3.36, p<0.001, Bonferroni-corrected p=0.005). No other pairwise comparisons reached statistical significance.

#### 3.4.3 Gender differences and pathways of care

Male patients were significantly more likely to be subjected to mechanical restraint during involuntary psychiatric hospitalization compared to female patients. Specifically, 12 out of 111 men (10.8%) experienced at least one episode of mechanical restraint, whereas this occurred in only 1 out of 89 women (1.1%). This difference was statistically significant (χ²(1)=7.63, p=0.006), and the result was confirmed by Fisher’s exact test (p=0.007). The odds of being restrained were therefore considerably higher for men, supporting the hypothesis that male patients are at greater risk of undergoing coercive measures.

In terms of treatment duration, women showed a slightly shorter average length of stay (mean 6.9 days, SD= ± 2.7) compared to men (mean 7.7 days, SD= ± 4.5). However, this difference was not statistically significant (Mann–Whitney U = 4794.000, p=0.833), and the effect size was negligible, indicating that sex did not meaningfully influence the duration of involuntary hospitalization.

The hypothesis that the duration of IPT varies according to the validation setting was partially supported. Overall differences across validation settings were statistically significant (Kruskal–Wallis test: χ²(4)=13.18, p=0.010). Descriptively, the longest mean duration was observed for cases validated by the Psychiatric Consultation Service (mean = 10.0 days, SD = ± 7.39), followed by Community Psychiatric Services (mean = 7.9 days, SD = ± 4.16), Emergency Departments (mean = 7.3 days, SD = ± 3.34), and the Psychiatric Diagnosis and Treatment Unit (mean = 6.5 days, SD = ± 2.44). The shortest duration was observed for cases validated in Other Services (mean = 3.9 days, SD = ± 2.30). *Post hoc* analyses using Dunn’s test indicated that cases validated in Other Services had significantly shorter IPT durations compared with Community Psychiatric Services (z=3.43, Bonferroni-corrected p=0.006) and Emergency Departments (z=3.12, Bonferroni-corrected p=0.018). No other pairwise comparisons remained statistically significant after correction for multiple testing. Overall, these findings suggest that IPT duration is broadly comparable across structured emergency, inpatient, and territorial pathways, whereas markedly shorter hospitalizations appear limited to a small subgroup of cases validated in residual or atypical service settings. Detailed results are shown in **Table 3**.

**Table 3:** Duration of involuntary psychiatric treatment (IPT) by validation setting.

Validation setting	N	Mean (days)	SD
Community psychiatric services	93	7.9	±4.2
Emergency departments	70	7.3	±3.3
Psychiatric Diagnosis and Treatment Unit	22	6.5	±2.4
Other services	8	3.9	±2.3
Psychiatric Consultation Service	4	10	±7.4
Dunn’s *Post Hoc* Comparison	z	p	pBonf
Psychiatric Consultation Service—Community psychiatric services	0.33	0.74	1
Psychiatric Consultation Service—Emergency departments	0.52	0.60	1
Psychiatric Consultation Service—Psychiatric Diagnosis and Treatment Unit	0.93	0.35	1
Psychiatric Consultation Service—Other services	2.34	0.02	0.2
Community psychiatric services—Emergency departments	0.62	0.54	1
Community psychiatric services—Psychiatric Diagnosis and Treatment Unit	1.42	0.16	1
Community psychiatric services—Other services	3.43	<0.001	0.006
Emergency departments—Psychiatric Diagnosis and Treatment Unit	0.98	0.33	1
Emergency departments—Other services	3.12	0.002	0.02
Psychiatric Diagnosis and Treatment Unit—Other services	2.24	0.03	0.25

Analyses are based on cases with complete data on IPT duration.

## 4 Discussion

The present study explored factors associated with IPT in the province of Reggio Emilia, within the national multicenter project promoted by SIEP. The analysis was guided by three main domains of hypotheses—service engagement and clinical history, diagnostic and clinical characteristics, and gender differences and care pathways. Findings provide a nuanced picture of how coercion is implemented in a local Italian context, while also contributing to the broader international debate on factors associated with, consequences, and alternatives to IPT.

International evidence consistently indicates that IPT is associated with a combination of clinical factors (acute psychosis, behavioral dyscontrol, substance use, prior coercion), social determinants (social isolation, marginalization, minority status), and organizational characteristics (bed availability, crisis alternatives, staff attitudes, continuity of care) (10, 13). Against this background, our local findings contribute by highlighting the role of prior IPT history in renewals, the gendered distribution of restraint, and the impact of validation pathways on IPT duration in an Italian province-wide catchment area.

### 4.1 Service engagement and clinical history

Our first set of hypotheses concerned the role of previous contact with mental health services, prior history of coercion, and the modality of admission (direct vs. conversion from voluntary). We expected that first-ever patients would experience longer IPTs, that prior IPT would predict renewal, and that conversions from voluntary admission would be shorter than direct compulsory admissions.

Results only partially supported these hypotheses. Contrary to expectations, there was no significant difference in IPT duration between first-ever patients and those already in care. This finding diverges from prior evidence suggesting that lack of service engagement predicts both higher risk and longer duration of compulsory hospitalization (26). In our context, organizational factors or legal safeguards may mitigate this effect. It is also plausible that the prompt involvement of community mental health services prevents delays in care transitions, thereby avoiding unnecessary prolongation of hospitalization. This would suggest that, once IPT is initiated, acute clinical severity and risk profiles, rather than service history or admission pathway, primarily determine duration (27).

By contrast, prior history of IPT emerged as a robust predictor of renewal. Patients with previous coercive experiences had a markedly higher probability of IPT prolongation compared to first−time cases. This recurrence effect is well documented: international studies consistently show that patients who have been coerced once are at higher risk of future coercion. For example, Lay et al. (15) found that a history of previous compulsory admissions increased the hazard of re−admission by approximately 1.8 times (and even more when the prior admission involved endangerment of others). Similarly, Zervakis et al. (28) showed that prior involuntary commitment was significantly associated with increased perceived coercion during subsequent voluntary admissions (rate ratio=1.6; p=0.05). Moreover, Martinez et al. (29) reported that past coercive experiences predict higher levels of perceived pressure and fear, and lower satisfaction in later hospitalizations, even when these are voluntary. Beyond the psychological and relational aftermath of prior coercion, Müller et al. (30) demonstrated that chronicity and prior involuntary treatment significantly predict earlier and more frequent coercive measures in psychiatric inpatient care. These findings suggest that, beyond clinical relapses, relational and trust barriers generated by previous involuntary interventions reinforce a cycle of exclusion and coercion.

Finally, the hypothesis that conversions from voluntary admission would be associated with shorter IPT was not supported. The absence of differences between converted and direct IPTs suggests that the clinical severity that leads to coercion outweighs admission pathway. Similar findings have been reported in German and Swiss samples, where conversions did not significantly alter hospitalization outcomes (6, 31). Taken together, these results highlight the complex relationship between service engagement and coercion: while engagement alone may not shorten duration once IPT is initiated, a history of coercion strongly increases the risk of renewed involuntariness, underlining the need for preventive, community-based approaches for high-risk patients.

### 4.2 Diagnostic and clinical characteristics

The second domain concerned diagnostic correlates. As hypothesized, diagnosis significantly influenced IPT duration, with schizophrenia spectrum and delusional disorders associated with the longest stays. These findings confirm a large body of evidence showing that psychotic disorders are the strongest predictor of both compulsory admission and prolonged coercion. Indeed, a meta-analysis by Walker et al. (13) reported that diagnosis of a psychotic disorder was associated with one of the highest risks of involuntary psychiatric admission (OR = 2.18, 95% CI 1.95–2.44). Additionally, Flammer and Steinert (32) found that, among non-organic diagnoses, psychotic disorders were the most important predictor of detention during hospitalization. These observations are consistent with findings from Iozzino et al. (16), who, in a systematic review and meta-analysis of violence in acute psychiatric wards, reported an increased risk of aggressive behavior among patients with psychotic disorders. While aggressive behavior often represents a trigger for coercive interventions, this association should be interpreted with caution, as it may reflect not only clinical features but also contextual, relational, and organizational factors influencing the use of compulsory measures.

The higher IPT duration among psychotic patients may reflect several converging factors: reduced insight and treatment adherence, risk of aggression, and complexity of stabilization. Moreover, previous studies show that clinicians tend to perceive psychosis as less amenable to consensual negotiation, thus lowering the threshold for coercion (6).

Interestingly, bipolar disorder and other diagnostic categories were associated with shorter IPT durations, and personality disorders showed the shortest IPT durations, although this difference reached statistical significance only in comparison with schizophrenia spectrum disorders. While our findings cannot be directly compared with duration-related data in other settings, they are in line with international evidence showing that affective disorders are less frequently managed under prolonged coercion (30). At the same time, some studies suggest that patients with borderline personality disorder may face high rates of short-term coercion (e.g., seclusion or restraint), often driven by crisis behavior. For example, a recent study in child and adolescent psychiatry reported that manic episodes/bipolar disorder (OR = 36.4) and personality disorders (OR = 15.2) were strongly associated with increased likelihood of seclusion or restraint (33). Our data, showing no significant difference in restraint across diagnostic groups in Reggio Emilia, suggest that in this context diagnosis mainly influences duration rather than the type of coercion.

The high proportion of schizophrenia spectrum diagnoses in our IPT cohort may differ from patterns reported in some countries, where affective disorders (including major depression) contribute substantially to involuntary admissions. This discrepancy likely reflects differences in service pathways, legal and clinical thresholds for IPT, and local crisis-management practices. In our setting, IPT appears to be more commonly activated during acute psychotic decompensations requiring urgent containment and stabilization, whereas severe depressive episodes may more often be managed through voluntary admissions or alternative pathways, unless accompanied by suicidality or psychotic features.

When IPT occurs in the context of major depressive disorder, it is most plausibly driven by acute suicidal risk, psychotic depression, or other high-risk clinical scenarios rather than by impaired decision-making per se. In the absence of structured data on suicidality, psychotic features, or imminent danger at admission, this interpretation remains inferential. Nevertheless, international literature consistently indicates that severe affective episodes accompanied by suicidality or psychotic symptoms represent common pathways to compulsory treatment (16).

Finally, these results underscore the importance of implementing early intervention services for psychosis, which have been shown to improve outcomes and, in some contexts, reduce the risk of involuntary treatment. For instance, randomized and review evidence indicates that specialized early intervention services enhance functioning, adherence, and patient satisfaction (34) and that structured crisis interventions such as joint crisis plans can reduce involuntary admissions (35). Developing such structured alternatives may help manage acute psychotic decompensation without resorting to prolonged IPT.

### 4.3 Gender differences and pathways of care

The third domain examined gender and care pathways, focusing on restraint use, duration, and setting of validation.

As hypothesized, male patients were significantly more likely to be mechanically restrained than females. This aligns with multiple studies demonstrating that male gender is a risk factor for coercive measures: in a recent multicenter study in Paris, male sex was independently associated with a higher likelihood of experiencing mechanical restraint (36). Similarly, Nawka et al. (37) found that physical restraint was used more frequently with male patients, while forced medication and seclusion were relatively more common among females. These findings may stem from gendered perceptions of aggression and risk—staff may perceive men as more dangerous, thus lowering the threshold for restraint. Ethically, this disparity is concerning; it indicates that gender stereotypes can color clinical decision-making and underscores the urgent need for trauma-informed, bias-aware training for healthcare professionals.

In contrast, the hypothesis that women would experience shorter IPT durations was not confirmed. Although the average duration was slightly lower for women, the difference was not statistically significant. This suggests that while gender impacts the likelihood of restraint, it does not strongly affect the length of compulsory hospitalization (38).

While international studies suggest that emergency settings may be associated with faster crisis resolution, our local findings indicate that, after correction for multiple comparisons, IPT duration is broadly comparable across emergency departments, inpatient units, and community psychiatric services. The absence of large differences across standard care pathways does not negate the role of organization but rather suggests a relatively homogeneous service culture within the province, likely shaped by shared clinical practices, legal safeguards, and integrated service governance. The only setting associated with significantly shorter IPT durations was a residual category of “other services”, suggesting that organizational context per se does not systematically determine duration, except in atypical validation pathways.

Similar patterns have been reported in other European emergency psychiatry settings, where male gender and psychotic spectrum diagnoses are consistently associated with higher use of coercive measures, while differences across care settings tend to diminish once organizational characteristics are taken into account (39).

### 4.4 Strengths and limitations

This study has several strengths. It is based on exhaustive data collection covering all IPT cases over a full year in a defined catchment area, ensuring completeness and minimizing bias. Integration into a national multicenter initiative (SIEP) allowed standardized procedures and comparability across sites. Unlike many studies relying solely on administrative data, we included clinical, demographic, and organizational variables, offering a multidimensional perspective. The study also followed rigorous ethical standards and used validated instruments with trained staff.

Nonetheless, important limitations restrict generalizability. The single-site design is the main weakness: findings from one unit cannot represent national practice given regional variability in IPT. Although the sample included all local cases, subgroup analyses suffered from small cell counts, reducing power. Missing contextual variables—such as socioeconomic status, family support, employment, staff ratios, and ward climate—limit explanatory depth. Patient and family perspectives were absent, despite coercion being primarily experienced as traumatic (5). Future studies should adopt participatory approaches to integrate these voices.

Outcome measures were restricted: while we assessed duration, renewal, and restraint, other forms of coercion (e.g., forced medication, seclusion) and long-term outcomes (e.g., relapse, functional recovery, trust) were not captured. This narrow focus risks portraying IPT as isolated rather than embedded in broader care trajectories (6, 30).

A further limitation is the absence of structured clinical indicators that commonly trigger involuntary treatment, such as imminent danger to self or others, suicidal attempts, intentions or plans, severity of psychotic symptoms, severe self-neglect, or aggressive behavior at admission. These factors are key drivers of compulsory pathways and may cut across diagnostic categories. Their unavailability prevents us from disentangling whether observed associations (e.g., diagnosis or validation setting) reflect underlying acute risk/severity profiles rather than service-level or organizational mechanisms.

Information on the presence or involvement of legal representatives or guardians was not collected, preventing any assessment of their potential impact on consent procedures, appeals, or coercive pathways. This represents an important legal and ethical dimension that should be addressed in future studies.

We were unable to compare the diagnostic distribution of involuntary psychiatric admissions with the diagnostic distribution of all psychiatric hospitalizations in the catchment area during the same period, due to the unavailability of comprehensive local denominator data. Such comparisons would be essential to determine whether specific diagnoses are over-represented among IPT cases relative to overall admissions and should be addressed in future multicenter and pooled analyses within the SIEP project.

Finally, some effect sizes were small or negligible, warranting cautious interpretation of statistically significant results.

## 5 Conclusions

This study provides evidence on involuntary psychiatric treatment (IPT) in an Italian local context. Schizophrenia spectrum and delusional disorders were associated with longer IPT durations, while a history of prior coercion was strongly associated with renewal. Male gender was linked to a higher likelihood of mechanical restraint but not to longer IPT duration. Differences in IPT duration across validation settings were observed; however, these were primarily driven by a small subgroup of cases validated in residual or atypical services, whereas emergency, inpatient, and community pathways showed broadly comparable durations. Overall, prior coercion history and organizational pathways appeared to outweigh demographic characteristics in shaping IPT-related outcomes, reinforcing the view that coercion is not merely a function of individual pathology but also of service organization (40, 41).

## Data Availability

The raw data supporting the conclusions of this article will be made available by the authors, without undue reservation.
